# Unraveling the Factors Associated With Digital Health Intervention Uptake: Cross-Sectional Study

**DOI:** 10.2196/63896

**Published:** 2025-12-09

**Authors:** Ilona Ruotsalainen, Mikko Valtanen, Riikka Kärsämä, Adil Umer, Hilkka Liedes, Suvi Parikka, Annamari Lundqvist, Kirsikka Aittola, Suvi Koivunen, Jussi Pihlajamäki, Anna-Leena Vuorinen, Jaana Lindström

**Affiliations:** 1 VTT Technical Research Centre of Finland Ltd Kuopio Finland; 2 Population Health Unit Finnish Institute for Health and Welfare Helsinki Finland; 3 Department of Mathematics and Statistics University of Turku Turku Finland; 4 VTT Technical Research Centre of Finland Ltd. Espoo Finland; 5 Institute of Public Health and Clinical Nutrition School of Medicine University of Eastern Finland Kuopio Finland; 6 Department of Medicine Endocrinology and Clinical Nutrition Kuopio University Hospital Kuopio Finland; 7 Unit of Health Sciences Faculty of Social Sciences Tampere University Tampere Finland

**Keywords:** digital health intervention, DHI, uptake, lifestyle, adoption, digital literacy, health equity, cross-sectional study, literacy, cross-sectional, socioeconomic, habits, e-services, questionnaire survey, regression model, Finland, healthy lifestyle, logistic regression model

## Abstract

**Background:**

Chronic noncommunicable diseases (NCDs) remain a leading health challenge worldwide, and reducing modifiable lifestyle risk factors is a key prevention strategy. Digital health interventions (DHIs) offer scalable, cost-effective tools to support healthy behaviors, but concerns persist about their equitable reach and uptake across population groups.

**Objective:**

This study aimed to examine how socioeconomic factors, health status, lifestyle behaviors, and attitudes and experiences related to the use of electronic services (e-services) are associated with the uptake of a DHI.

**Methods:**

In this cross-sectional study, we invited (through mail or SMS) a subgroup of 6978 participants aged 20-74 years from the population-based Healthy Finland survey to take part in a DHI. The DHI, delivered via the web-based BitHabit app, aimed to support the adoption of healthy lifestyle habits. Uptake was defined as successful registration, agreeing to the terms of use, and accepting the invitation to participate. Predictor variables were drawn from national registry and self-reported survey data and included socioeconomic status, health indicators, lifestyle behaviors, and attitudes and experiences related to the use of e-services. Adjusted logistic regression models were used to identify significant predictors of DHI uptake.

**Results:**

Of the final sample of 6975 participants, 1287 (18.5%) started using the DHI. Uptake was significantly higher among women (adjusted odds ratio [aOR] 1.69, 95% CI 1.49-1.93), middle-aged individuals (aOR 1.47, 95% CI 1.21-1.79), and those with higher income (aORs 1.76-1.97, 95% CIs 1.37-2.59) and more years of education (aOR 1.10, 95% CI 1.08-1.12). Healthier lifestyle indicators, including better diet quality (aOR 1.07, 95% CI 1.04-1.10), less frequent smoking or nonsmoking (aORs 1.59-2.29, 95% CIs 1.08-3.12), sleep (aOR 0.58, 95% CI 0.37-0.86), higher functional capacity (aOR 1.06, 95% CI 1.02-1.11), and good overall current health (aOR 1.46, 95% CI 1.15-1.89), were associated with increased likelihood of DHI uptake. The strongest predictors were related to the use of e-services: Individuals who used e-services (aORs 2.48-6.08, 95% CIs 1.19-11.92) reported higher competence to use e-services (aORs 2.00-4.10, 95% CIs 1.44-5.92), had low concerns about data security (aORs 1.37-1.76, 95% CIs 1.03-2.33), believed in the benefits of digital services (aOR 1.04, 95% CI 1.02-1.05), and had better internet connections had higher odds of uptake.

**Conclusions:**

Our findings show that DHI uptake is associated with socioeconomic status, health and lifestyle factors, and, especially, individuals’ experience and attitudes toward e-services. Individuals with lower education levels, lower income, and poorer health and lifestyle habits are less likely to adopt DHIs, raising concerns about potential digital health inequities. These results underscore the need for targeted strategies to reduce barriers and ensure more equitable reach and engagement in future DHI implementations.

## Introduction

Chronic noncommunicable diseases (NCDs) represent a significant health challenge worldwide, imposing an increasing economic burden on health care services and resulting in premature deaths [1,2]. To reduce the burden of NCDs, reducing modifiable lifestyle risk factors has been highlighted as one of the most important actions worldwide [3]. Adopting a healthy lifestyle can potentially reduce the risk of developing common chronic diseases by up to 80% [4]. There is a pressing need to develop strategies that promote the widespread adoption of healthy lifestyle habits, aiming to achieve impactful results at a large scale.

Digital health interventions (DHIs) offer a viable way to support a healthy lifestyle and hold promise as a low-cost means of delivering interventions to large population groups. However, DHIs can be effective only if they reach the target population, especially those who need them most. Ensuring that DHIs reach their intended users requires awareness of the potential barriers to uptake, which has been broadly defined as the initial step of registering for, downloading, or otherwise beginning to use a DHI [5,6]. This is particularly important in the context of NCDs, which disproportionately affect populations with lower socioeconomic status and limited access to health care services [7,8]. DHIs have the potential to improve access to preventive care and support individuals in managing their health, but to truly make a difference, it is essential to remove barriers that could otherwise deepen existing health inequalities [9]. Currently, there is a lack of comprehensive understanding regarding the factors influencing DHI uptake. Identifying these factors will facilitate the customization of future interventions and recruitment strategies, thereby enhancing adoption rates and promoting digital equity.

Digital health promotion is an emerging field, and therefore, research on DHIs remains scarce [10]. Much of the existing literature has concentrated on health apps, which often lack a defined duration, evidence-based modules, and explicit behavior change techniques that are typically built into DHIs [11,12]. These studies have shown that females [6,13,14], young or middle-aged individuals [6,13,14], and those with higher education levels [6,13,15] and higher income [6,15,16] tend to be more frequent users of health apps. Additionally, conflicting findings exist regarding the relationship between overall health [15,17,18], lifestyle habits [13,15,17,18], and health app usage. Recent research has also suggested that additional factors, such as perceived usefulness and privacy concerns, might play a significant role in influencing the adoption of health apps and digital health services [19-21]. Perceived usefulness has been associated with greater acceptance and intention to use digital health services across age groups [19,20]. In contrast, concerns regarding privacy and data security, particularly among older adults, can reduce usage intentions and act as barriers to adoption [19-22].

Studying health app users, although informative, does not offer comprehensive insights into potential barriers to DHI uptake. First, the lack of comparative analysis between adopters (individuals who start using DHIs) and nonadopters raises questions about whether observed characteristics reflect a higher uptake probability or the characteristics of the populations to which the apps were advertised. Second, the usage of health apps itself may influence individuals’ lifestyle habits and health [23], introducing a potential bias and thereby complicating the interpretation of these findings in relation to DHI adoption. Lastly, usage is also influenced by other factors, such as user experience, which may be affected by the app quality [24].

Here, our aim was to study factors predicting DHI uptake to identify potential groups that may be less likely to start using DHIs. We examined whether socioeconomic factors, self-reported health and lifestyle habits, or habitual use of electronic services (e-services) predicts DHI uptake. Understanding these factors will help identify population groups that are underrepresented in DHIs and find alternative approaches for them. These findings will also aid in integrating potential barriers into the formulation of recruitment strategies and the development of DHIs themselves.

## Methods

### Participants and Study Design

This cross-sectional study was a substudy of the Healthy Finland population survey, the methodology of which is described in detail elsewhere [25,26]. In this survey, a questionnaire on health, well-being, and service use was sent to randomly selected individuals over the age of 20 years, representing the entire adult population of Finland. For our study, Finnish-speaking individuals aged 20-74 years who had completed the Healthy Finland questionnaire between September 2022 and the end of 2022 were considered eligible, excluding those randomly selected for the health examination component to prevent potential influence of the DHI on examination results. Among this eligible population (N=10,207), an SMS invitation was sent to all individuals with a known phone number (n=4978, 48.8%). Additionally, 2000 (19.6%) individuals from the remaining eligible population were sampled based on 5-year age groups to receive a letter invitation. Three participants asked their data to be removed, resulting in a final sample size of 6975 (99.9%) individuals. The sample size was chosen to detect small effects in logistic regression, with a statistical power of 0.8 and a type I error of 0.05, assuming that approximately 10%-15% of invitees would adopt the DHI. Based on this, a minimum total sample size of 2538 participants was required.

Participants were offered the opportunity to use a DHI app (BitHabit) for 3 months [27]. The invitation letter included a web address, and the SMS message contained a direct link to the project’s web page (hosted by the Finnish Institute for Health and Welfare), where participants could find information regarding the study, a link to the BitHabit app, and brief instructions on how to get started with the app.

### Ethical Considerations

Ethical approval for the study was obtained from the research ethics committee of the Finnish Institute for Health and Welfare (THL/5335/6.02.01/2022). The approval covered all the procedures, data, and analysis presented in this study. Participation in the Healthy Finland survey was voluntary, and participants were informed that completing and returning the questionnaire constituted consent to participate. In addition, digital informed consent was obtained following BitHabit registration. Participants were informed of their right to decline or withdraw participation at any time. All data were pseudonymized before analysis, and the study was conducted in accordance with the General Data Protection Regulation (GDPR) and applicable Finnish data protection legislation and the analysis conducted in a secure analysis environment. No compensation was provided to participants.

### The Digital Health Intervention App (BitHabit)

The BitHabit app was originally developed to support the formation of healthy lifestyle habits in adults at increased risk of type 2 diabetes [27,28]. The app includes a broad selection of small health-promoting actions (“habits”) in 14 different categories related to physical activity, diet, sleep, stress management, positive mood, smoking, and alcohol consumption. It is a web-based app that can be used on a mobile phone, tablet, or computer. To log into the app, participants had to provide a phone number and a user ID that was specified either in the letter or in the SMS message. Uptake of the app was defined as the process of registering for the BitHabit app with a phone number and user ID, agreeing to the terms of use, and, subsequently accepting the invitation to participate. No additional confirmation of refusal was collected from those who did not register.

### Predictor Variables

We examined predictors from the following categories: socioeconomics, health, lifestyle habits, and the use of e-services. All predictor variables except age and sex (assigned at birth), which were obtained from the Finnish National Population Register, were derived from the Healthy Finland questionnaire and were thus self-reported. The survey was conducted approximately 2-4 months prior to sending invitations to take part in the DHI substudy, and the BitHabit app was offered only after the survey responses had been received. Following the invitation, participants had approximately 4 weeks to begin using the app. All questions and the answer options are presented in Multimedia Appendix 1.

#### Socioeconomics

Socioeconomic variables included age, sex, education (years of education, including primary and comprehensive school), household income (€ per year before taxes), and employment status. Household income was coded as a five-category ordinal variable: <€15,000 (<US $17,437; calculated at an exchange rate of €1=US $1.16 approximately), €15,001-35,000 (US $17,438-$40,686), €35,001-55,000 (US $40,687-$63,935), €55,001-75,000 (US $63,936-$87,184), and >€75,000 (>US $87,184). Age was categorized into four groups: 20-34, 35-49, 50-64, and 65-74 years.

#### Health

The BMI was calculated based on self-reported height and weight. Other health-related predictor variables included functional capacity, ability to work, current health status, prevalence of (any) long-term illness, health care service use (number of visits during the past 12 months), perceived ability to learn, and perceived limitations due to health problems. Functional capacity was calculated as a summary score for five questions on the abilities to perform the following activities: run for about 100 m, walk for about 500 m without stopping to rest, see ordinary newspaper print, hear what is said in a conversation between several people, and walk up one flight of stairs without stopping to rest. Each subquestion had four choices (yes, with no problem [3 points]; yes, with some difficulty [2 points]; yes, but with great difficulty [1 point]; no, I cannot [0 points]). A higher summary score indicated better functional capacity.

#### Lifestyle Habits

Lifestyle-related information included physical activity, sleep, smoking, and diet quality. Participants were divided into two groups based on whether they achieved the Finnish physical activity recommendations [29]. Sleep adequacy categories were created based on the question, “Do you get enough sleep?” The diet quality score was calculated following the method presented by Lindström et al [30], and the details of the scoring are in the *Diet Quality Scoring* section in Multimedia Appendix 1.

#### Use of e-Services

The following predictors relating to the use of e-services were considered: independence of e-service use, competence to use e-services, accessibility of e-services, concerns about data security, poor internet connections, and perceived benefits of e-services. The “perceived benefits of e-services” score was a summary measure calculated based on participants’ level of agreement with six claims: e-services (1) help me assess the need for services, (2) support me in finding and choosing the most suitable services, (3) make it easier for me to use the services regardless of where I am and when, (4) make it easier for me to collaborate with professionals, (5) help me take an active role in looking after my own health and welfare, and (6) help me take care of the health, welfare, and functional capacity of family or friends. Each claim had five categories (completely agree, somewhat agree, neither agree nor disagree, somewhat disagree, strongly disagree). A higher score indicated that the participant perceived e-services as more beneficial.

### Statistical Analysis

Logistic regression models were used to assess the association between the predictor variables and the uptake of the BitHabit DHI. The results were presented as adjusted odds ratios (aORs) with 95% CIs. Models examining variables related to health, lifestyle, and use of e-services were adjusted by contact method (SMS or letter), age, sex, education, and income. Regarding socioeconomic factors, models for age, sex, and contact method were adjusted by each other; models for income, BMI, and employment status by education, contact method, age, and sex; and the model for education by contact method, age, and sex. These covariates were selected a priori as background variables likely to be confounders of the predictor-outcome relationship based on the previous literature. The only exception was contact method (SMS vs letter), which was included to adjust for potential confounding introduced by the recruitment strategy: letter invitations were sampled by age to ensure representation across age groups, potentially correlating with both participant characteristics and DHI uptake. Participants with missing data (missing answers) were excluded from the analyses, and a complete-case analysis was performed. The amount of missing data for each predictor variable is presented in the *Results* section.

The overall significance of the associations between the categorical predictors and the outcome was assessed with likelihood ratio tests (LRTs), for which a false discovery rate (FDR)–corrected *P* value of <.05 was determined to indicate a significant association. R software version 4.3.1 (R Foundation for Statistical Computing [31]) was used to perform all statistical analyses. The reporting of this study followed the STROBE (Strengthening the Reporting of Observational Studies in Epidemiology) guidelines for reporting observational studies [32].

## Results

### Baseline Information

The final sample of 6975 participants, 1287 (18.5%) started using the BitHabit app (“adopters”). The uptake proportion was 16.2% (806/4975) among those who were invited via SMS and 24.1% (481/2000) among those with a letter invitation. Those invited via SMS were less likely to start using the app than those invited via mail (aOR 0.65, 95% CI 0.55-0.75). Background information for the entire study sample and separately for app adopters and nonadopters is presented in Tables 1-4.

**Table 1:** Socioeconomic characteristics of the study sample.^a^

Characteristics			Total sample (N=6975)	Adopters (n=1287)		Nonadopters (n=5688)
**Age (years), n (%)**						
	20-34	1229 (17.6)		250 (19.4)	979 (17.2)	
	35-49	1254 (18.0).		303 (23.5)	951 (16.7)	
	50-64	2066 (29.6)		361 (28.0)	1705 (30.0)	
	65-74	2426 (34.8)		373 (29.0)	2053 (36.1)	
**Sex, n (%)**						
	Female	3975 (57.0)		868 (67.4)	3107 (54.6)	
	Male	3000 (43.0)		419 (32.6)	2581 (45.4)	
**Education (years)**						
	Mean (SD)	14.0 (3.6)		15.2 (3.4)	13.8 (3.6)	
	Missing, n (%)	95 (1.4)		10 (0.8)	85 (1.5)	
**Annual household income (€; US $^b^), n (%)**						
	<15,000; <17,437	761 (10.9)		94 (7.3)	667 (11.7)	
	15,001-35,000; 17,438-40,686	1913 (27.4)		266 (20.7)	1647 (29.0)	
	35,001-55,000; 40,687-63,935	1916 (27.5)		380 (29.5)	1536 (27.0)	
	55,001-75,000; 63,936-87,184	1171 (16.8)		264 (20.5)	907 (15.9)	
	>75,000; >87,184	1119 (16.0)		272 (21.1)	847 (14.9)	
	Missing, n (%)	95 (1.4)		11 (0.9)	84 (1.5)	
**Employment status, n (%)^c^**						
	Employed full-time	1798 (44.2)		389 (51.1)	1409 (42.6)	
	Employed part-time	211 (5.2)		41 (5.4)	170 (5.1)	
	Retired	1463 (35.0)		228 (30.0)	1235 (37.4)	
	On a disability pension or rehabilitation benefit	161 (4.0)		24 (3.2)	137 (4.1)	
	Part-time retirement	27 (0.7)		1 (0.1)	26 (0.8)	
	Unemployed	169(4.2)		36 (4.7)	133 (4.0)	
	Family leave or stay-at-home parent	67(1.6)		19 (2.5)	48 (1.5)	
	Other	123(3.0)		21 (2.8)	102 (3.1)	
	Missing, n (%)	48 (1.2)		2 (0.3)	46 (1.4)	

^a^Data are reported as the mean (SD) for continuous variables and as n (%) for categorical variables.

^b^An exchange rate of €1=US $1.16 approximately was applied.

^c^The number of participants was as follows: total, N=4067; adopters, n=761; and nonadopters, n=3306.

**Table 2:** Health-related characteristics of the study sample.^a^

Characteristics		Total sample (N=6975)	Adopters (n=1287)	Nonadopters (n=5688)
**BMI (kg/m^2^)**				
	Mean (SD)	27.4 (5.3)	27.5 (5.5)	27.3 (5.2)
	Missing, n (%)	75 (1.1)	6 (0.5)	69 (1.2)
**Functional capacity**				
	Mean (SD)	13.8 (2.0)	14.1 (1.5)	13.7 (2.1)
	Missing, n (%)	144 (2.1)	15 (1.2)	129 (2.3)
**Ability to work**				
	Completely unable to work	350 (5.0)	49 (3.8)	301 (5.3)
	Partially unable to work	1688 (24.2)	247 (19.2)	1441 (25.3)
	Completely fit for work	4878 (69.9)	986 (76.6)	3892 (68.4)
	Missing, n (%)	59 (0.8)	5 (0.4)	54 (0.9)
**Current health status**				
	Poor or fairly poor	676 (9.7)	83 (6.4)	593 (10.4)
	Average	1740 (24.9)	278 (21.6)	1462 (25.7)
	Good or fairly good	4520 (64.8)	925 (71.9)	3595 (63.2)
	Missing, n (%)	39 (0.6)	1 (0.1)	38 (0.7)
**Long-term illnesses**				
	Yes	3937 (56.4)	713 (55.4)	3224 (56.7)
	No	2932 (42.0)	562 (43.7)	2370 (41.7)
	Missing, n (%)	106 (1.5)	12 (0.9)	94 (1.7)
**Health care visits**				
	Mean (SD)	5.7 (7.4)	5.8 (6.6)	5.6 (7.6)
	Missing, n (%)	655 (9.4)	125 (9.7)	530 (9.3)
**Memory and learning**				
	Poorly or very poorly	277 (4.0)	34 (2.6)	243 (4.3)
	Adequately	1779 (25.5)	249 (19.3)	1530 (26.9)
	Well or very well	4864 (69.7)	1001 (77.8)	3863 (67.9)
	Missing, n (%)	55 (0.8)	3 (0.2)	52 (0.9)
**Limitations due to health problems**				
	Severely limited	384 (5.5)	51 (4.0)	333 (5.9)
	Limited but not severely	2424 (34.8)	413 (32.1)	2011 (35.4)
	Not limited at all	4032 (57.8)	803 (62.4)	3229 (56.8)
	Missing, n (%)	135 (1.9)	20 (1.6)	115 (2.0)

^a^Data are reported as the mean (SD) for continuous variables and as n (%) for categorical variables.

**Table 3:** Lifestyle-related characteristics of the study sample.^a^

Characteristics			Total sample (N=6975)		Adopters (n=1287)		Nonadopters (n=5688)
**Diet quality**							
	Mean (SD)	8.1 (2.5)		8.5 (2.5)		7.9 (2.5)	
	Missing, n (%)	193 (2.8)		31 (2.4)		162 (2.8)	
**Physical activity**							
	Did not achieve recommended level	4122 (59.1)		741 (57.6)		3381 (59.4)	
	Achieved recommended level	2530 (36.3)		515 (40.0)		2015 (35.4)	
	Missing, n (%)	323 (4.6)		31 (2.4)		292 (5.2)	
**Enough sleep**							
	Yes, almost always or often	5324 (76.3)		991 (77.0)		4333 (76.2)	
	Rarely or hardly ever	1315 (18.9)		261 (20.3)		1054 (18.5)	
	Not sure	286 (4.1)		29 (2.3)		257 (4.5)	
	Missing, n (%)	50 (0.7)		6 (0.4)		44 (0.8)	
**Smoking**							
	Daily	675 (9.7)		56 (4.4)		619 (10.9)	
	Occasionally	419 (6.0)		64 (5.0)		355 (6.2)	
	Not at all	3137 (45.0)		586 (45.5)		2551 (44.8)	
	Have never smoked	2548 (36.5)		548 (42.6)		2000 (35.2)	
	Missing, n (%)	196 (2.8)		33 (2.5)		163 (2.9)	

^a^Data are reported as the mean (SD) for continuous variables and as n (%) for categorical variables.

**Table 4:** E-service^a^ use–related characteristics of the study sample.^b^

Characteristics			Total sample (N=6975)		Adopters (n=1287)		Nonadopters (n=5688)
**Independence of e-service use**							
	Do not use	447 (6.4)		11 (0.9)		436 (7.7)	
	Use with help or someone else uses on my behalf	306 (4.4)		20 (1.5)		286 (5.0)	
	Use independently	6177 (88.6)		1252 (97.3)		4925 (86.6)	
	Missing, n (%)	45 (0.6)		4 (0.3)		41 (0.7)	
**Competence to use online services**							
	No competence	208 (3.0)		6 (0.5)		202 (3.6)	
	Low competence	550 (7.9)		37 (2.9)		513 (9.0)	
	Moderate competence	2076 (29.7)		268 (20.8)		1808 (31.8)	
	High competence	1976 (28.3)		446 (34.6)		1530 (26.8)	
	Very high competence	2103 (30.2)		529 (41.1)		1574 (27.7)	
	Missing, n (%)	62 (0.9)		1 (0.1)		61 (1.1)	
**E-services** **not accessible to me**							
	Completely or somewhat agree	831 (11.9)		118 (9.2)		713 (12.5)	
	Neither agree nor disagree	1583 (22.7)		274 (21.3)		1309 (23.0)	
	Strongly or somewhat disagree	4022 (57.7)		838 (64.1)		3184 (56.0)	
	Missing, n (%)	539 (7.7)		57 (4.4)		482 (8.5)	
**Concerned about data security**							
	Completely agree	724 (10.3)		80 (6.2)		644 (11.3)	
	Somewhat agree	1441 (20.7)		255 (19.8)		1186 (20.9)	
	Neither agree nor disagree	1242 (17.8)		203 (15.8)		1039 (18.3)	
	Somewhat disagree	1392 (20.0)		310 (24.1)		1082 (19.0)	
	Strongly disagree	1882 (27.0)		415 (32.2)		1467 (25.8)	
	Missing, n (%)	294 (4.2)		24 (1.9)		270 (4.7)	
**Poor internet connections**							
	Completely or somewhat agree	727 (10.4)		111 (8.6)		616 (10.8)	
	Neither agree nor disagree	1050 (15.1)		151 (11.7)		899 (15.8)	
	Strongly or somewhat disagree	4820 (69.1)		996 (77.4)		3824 (67.2)	
	Missing, n (%)	378 (5.4)		29 (2.3)		349 (6.1)	
**Benefits of digital services**							
	Mean (SD)	16.4 (5.1)		17.4 (4.7)		16.1 (5.2)	
	Missing, n (%)	465 (6.7)		54 (4.2)		411 (7.2)	

^a^e-service: electronic service.

^b^Data are reported as the mean (SD) for continuous variables and as n (%) for categorical variables.

### Predictor Variables Associated With DHI Uptake

Factors associated with the uptake of the BitHabit app were studied in four domains: socioeconomics, health, lifestyle habits, and use of e-services. The aORs from all the models, along with associated *P* values and 95% CIs, are listed here, while details of the adjustments for each variable are listed in *Methods* section.

#### Socioeconomics

**Figure 1:**
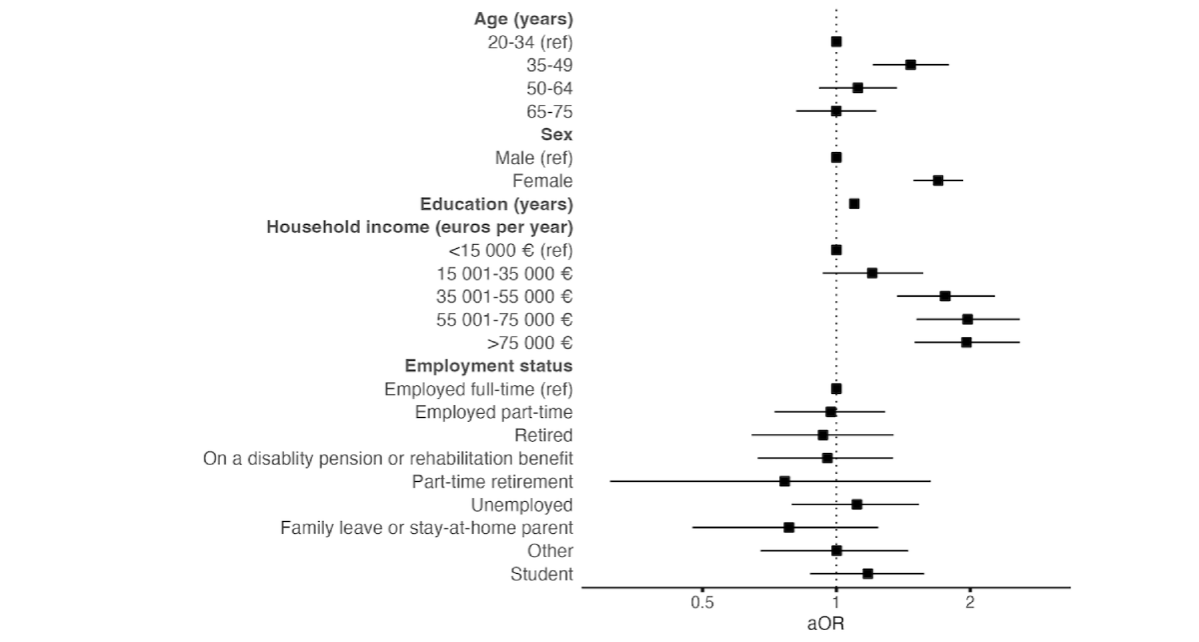
Forest plot showing aORs and 95% CIs for socioeconomic factors. Each aOR is adjusted for relevant covariates, as detailed in the *Statistical Analysis* section. aOR: adjusted odds ratio; ref: reference.

Age, sex, education, and household income demonstrated a significant association with uptake of the BitHabit DHI (Figure 1 and Table 5). Odds of adopting the DHI were higher for individuals aged 35-49 years compared to the reference group of 20-34 years (aOR 1.47, 95% CI 1.21-1.79). The two older age groups (50-64 and 65-74 years) showed no significant difference from the reference group. Higher educational attainment was associated with greater odds of DHI uptake (aOR 1.10, 95% CI 1.08-1.12). Similarly, higher income levels were associated with increased uptake, though the two highest categories had comparable estimates (€35,001-55,000: aOR 1.76, 95% CI 1.37-2.27; €55,001-75,000: aOR 1.97, 95% CI 1.51-2.59; >€75,000: aOR 1.96, 95% CI 1.50-2.59). In addition, women were more likely than men to adopt the DHI (aOR 1.69, 95% CI 1.49-1.93), whereas employment status was not significantly associated with DHI uptake.

**Table 5:** Logistic regression and LRT^a^ results for socioeconomic predictor variables.

Predictor variables			LRT				Logistic regression		
		*χ*^2^ (*df*)		FDR^b^-adjusted *P* value		aOR^c^ (95% CI)		*P* value (group comparison)	
**Age (years)**			**22.37 (3)**		**<.001**		—^d^		—
	20-34 (reference)	—		—		—		—	
	35-49	—		—		1.47 (1.21-1.79)		<.001	
	50-64	—		—		1.12 (0.91-1.37)		.28	
	65-75	—		—		1.00 (0.81-1.22)		.99	
**Sex**			**66.75 (1)**		**<.001**		—		—
	Male (reference)	—		—		—		—	
	Female	—		—		1.69 (1.49-1.93)		<.001	
Education (years)			98.8 (1)		<.001		1.10 (1.08-1.12)		
**Annual household income (€; US $^e^)**			**50.19 (4)**		**<.001**		—		—
	<15,000; <17,437 (reference)	—		—		—		—	
	15,001-35,000; 17,438-40,686	—		—		1.20 (0.93-1.57)		.16	
	35,001-55,000; 40,687-63,935	—		—		1.76 (1.37-2.27)		<.001	
	55,001-75,000; 63,936-87,184	—		—		1.97 (1.51-2.59)		<.001	
	>75,000; >87,184	—		—		1.96 (1.50-2.59)		<.001	
**Employment status**			**3.6 (8)**		**.89**		—		—
	Employed full-time (reference)	—		—		—		—	
	Employed part-time	—		—		0.97 (0.73-1.29)		.85	
	Retired	—		—		0.93 (0.64-1.34)		.71	
	On a disability pension or rehabilitation benefit	—		—		0.95 (0.67-1.34)		.79	
	Part-time retirement	—		—		0.77 (0.31-1.63)		.52	
	Unemployed	—		—		1.11 (0.79-1.53)		.53	
	Family leave or stay-at-home parent	—		—		0.78 (0.47-1.24)		.32	
	Other	—		—		1.00 (0.68-1.45)		.99	
	Student	—		—		1.18 (0.87-1.58)		.28	

^a^LRT: likelihood ratio test.

^b^FDR: false discovery rate.

^c^aOR: adjusted odds ratio.

^d^Not applicable.

^e^An exchange rate of €1=US $1.16 approximately was applied.

#### Health

**Figure 2:**
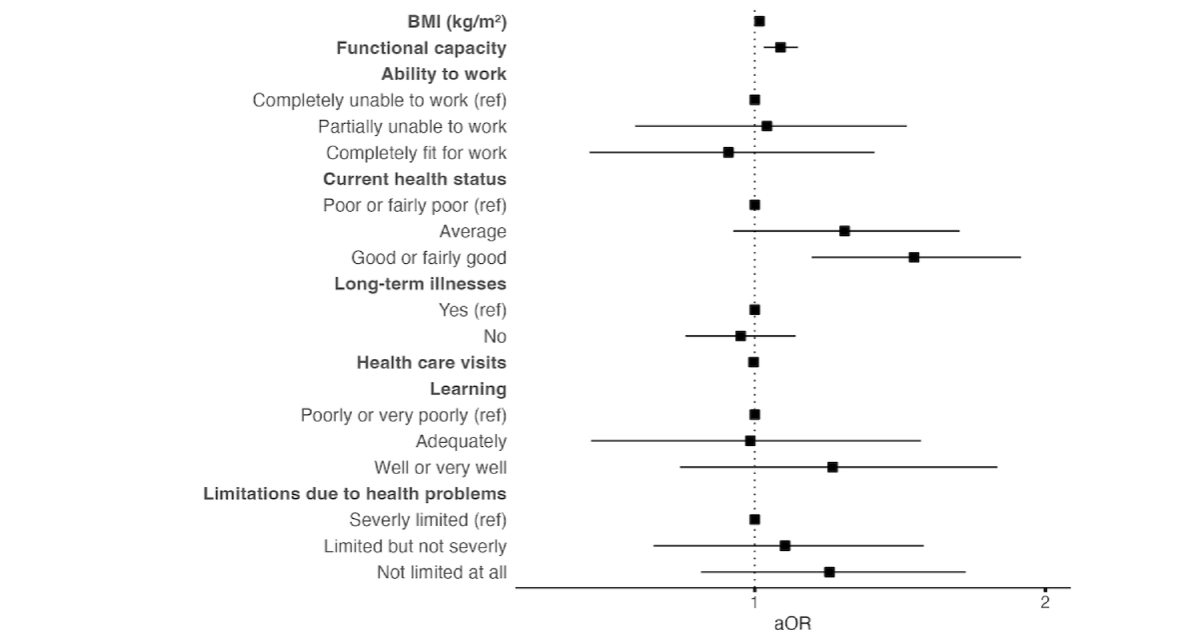
Forest plot showing aORs and 95% CIs for health-related predictors. Each aOR is adjusted for relevant covariates, as detailed in the *Statistical Analysis* section. aOR: adjusted odds ratio; ref: reference.

Among the health-related predictors, functional capacity and current health status showed significant associations with DHI uptake (Figure 2 and Table 6). The odds of adopting the DHI were higher for individuals with better functional status (aOR 1.06, 95% CI 1.02-1.11) and better current health (good or fairly good compared to poor: aOR 1.46, 95% CI 1.15-1.89). In contrast, none of the other health indicators, including the BMI, ability to work, ability to learn, the presence of long-term illnesses, the number of health care visits during the past 12 months, and limitations due to health problems, were significantly associated with uptake of the DHI.

**Table 6:** Logistic regression and LRT^a^ results for health-related predictor variables.

Predictor variables			LRT				Logistic regression		
		*χ*^2^ (*df*)		FDR^b^-adjusted *P* value	aOR^c^ (95% CI)			*P* value (group comparison)	
BMI (kg/m^2^)			3.6 (1)	.08		1.01 (1.00-1.02)			—^d^
Functional capacity			9.27 (1)	.005		1.06 (1.02-1.11)			—
**Ability to work**			**1.16 (2)**	**.61**		—			—
	Completely unable to work (reference)	—		—	—			—	
	Partially unable to work	—		—	1.03 (0.75-1.44)			.86	
	Completely fit for work	—		—	0.94 (0.67-1.33)			.72	
**Current health status**			**11.99 (2)**	**.005**		—			—
	Poor or fairly poor (reference)	—		—	—			—	
	Average	—		—	1.24 (0.95-1.63)			.12	
	Good or fairly good	—		—	1.46 (1.15-1.89)			.003	
**Long-term illnesses**			**0.25 (1)**	**.64**		—			—
	Yes (reference)	—		—	—			—	
	No	—		—	0.97 (0.85-1.10)			.62	
**Health care visits**			**0.41 (1)**	**.60**		**1.00 (0.99-1.01)**			—
**Ability to learn**			**6.24 (2)**	**.06**		—			—
	Poorly or very poorly (reference)	—		—	—			—	
	Adequately	—		—	0.99 (0.68-1.49)			.96	
	Well or very well	—		—	1.20 (0.84-1.78)			.33	
**Limitations due to health problems**			**3.06 (2)**	**.26**		—			—
	Severely limited (reference)	—		—	—			—	
	Limited but not severely	—		—	1.07 (0.79-1.50)			.66	
	Not limited at all	—		—	1.19 (0.88-1.65)			.27	

^a^LRT: likelihood ratio test.

^b^FDR: false discovery rate.

^c^aOR: adjusted odds ratio.

^d^Not applicable.

#### Lifestyle Habits

**Figure 3:**
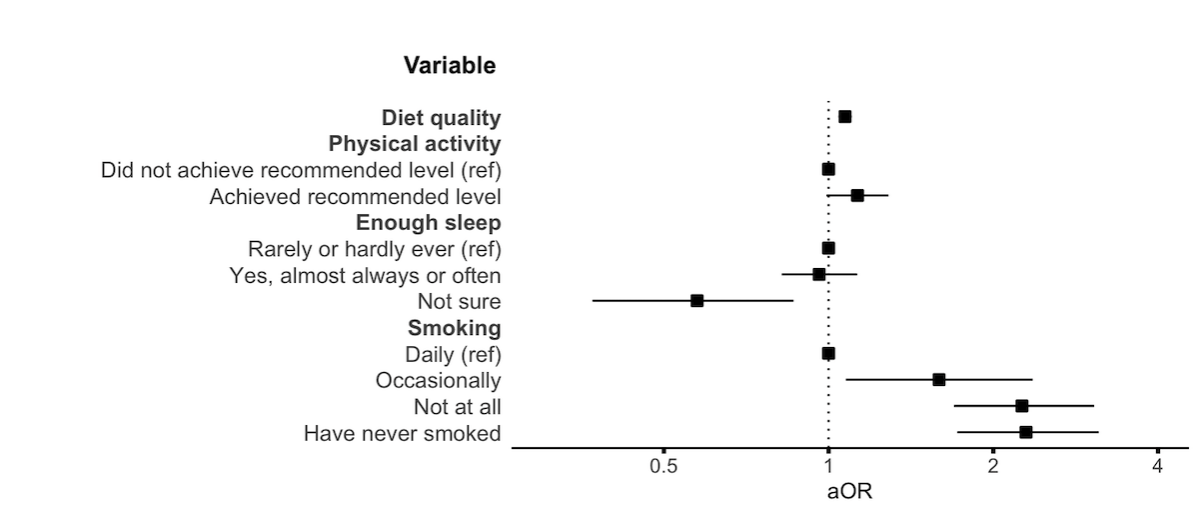
Forest plot showing aORs and 95% CIs for lifestyle habits. Each aOR is adjusted for relevant covariates, as detailed in the *Statistical Analysis* section. aOR: adjusted odds ratio; ref: reference.

Diet quality, smoking, and sleeping were associated with the uptake of the DHI (Figure 3 and Table 7). Participants with better diet quality (aOR 1.07, 95% CI 1.04-1.10) had higher odds of DHI uptake. Similarly, smoking behavior showed a clear gradient: Compared to daily smokers, those who smoked occasionally (aOR 1.59, 95% CI 1.08-2.36), did not smoke at the time of the survey (aOR 2.26, 95% CI 1.69-3.06), or had never smoked (aOR 2.29, 95% CI 1.72-3.12) had higher odds of adopting the DHI. Sleeping enough was also a significant predictor; however, no meaningful difference was estimated between those sleeping mostly enough and the reference category of those sleeping mostly not enough. Instead, participants who were unsure of whether they slept enough had significantly lower odds of DHI uptake compared to the reference category (aOR 0.58, 95% CI 0.37-0.86). In contrast, achieving physical activity recommendations was not significantly associated with uptake of the DHI.

**Table 7:** Logistic regression and LRT^a^ results for lifestyle-related predictor variables.

Predictor variables			LRT				Logistic regression		
		*χ*^2^ (*df*)		FDR^b^-adjusted *P* value		aOR^c^ (95% CI)		*P* value (group comparison)	
Diet quality			23.67 (1)		<.001		1.07 (1.04-1.10)		—^d^
**Physical activity**			**3.34 (1)**		**.09**		—		—
	Did not achieve recommended level (reference)	—		—		—		—	
	Achieved recommended level	—		—		1.13 (0.99-1.29)		.07	
**Enough sleep**			**7.54 (2)**		**.04**		—		—
	Rarely or hardly ever (reference)	—		—		—		—	
	Yes, almost always or often	—		—		0.96 (0.82-1.13)		.62	
	Not sure	—		—		0.58 (0.37-0.86)		.01	
**Smoking**			**41.76 (3)**		**<.001**		—		—
	Daily (reference)	—		—		—		—	
	Occasionally	—		—		1.59 (1.08-2.36)		.02	
	Not at all	—		—		2.26 (1.69-3.06)		<.001	
	Have never smoked	—		—		2.29 (1.72-3.12)		<.001	

^a^LRT: likelihood ratio test.

^b^FDR: false discovery rate.

^c^aOR: adjusted odds ratio.

^d^Not applicable.

#### Use of e-Services

**Figure 4:**
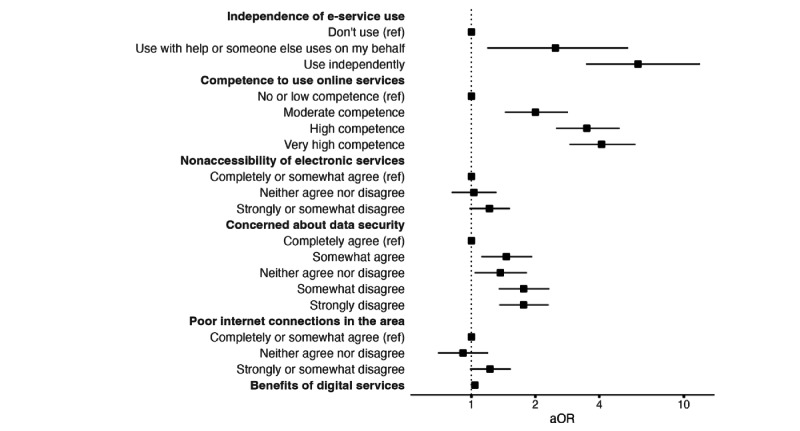
Forest plot showing aORs and 95% CIs for e-service use–related factors. Each aOR is adjusted for relevant covariates, as detailed in the *Statistical Analysis* section. aOR: adjusted odds ratio; e-service: electronic service; ref: reference.

All examined factors related to the use of e-services, except the perceived accessibility of e-services, showed a significant association with DHI uptake (Figure 4 and Table 8). Individuals who reported using e-services had notably higher odds of adopting the DHI. Compared to those who did not use e-services at all, participants who used them with assistance had more than twice the odds of DHI uptake (aOR 2.48, 95% CI 1.19-5.47), while those who used them independently had over six times the odds of DHI uptake (aOR 6.08, 95% CI 3.46-11.92). Competence in using online services also showed a strong relationship: Those with moderate competence had an aOR of 2.00 (95% CI 1.44-2.85), those with high competence had an aOR of 3.49 (95% CI 2.50-4.98), and those with very high competence had an aOR of 4.10 (95% CI 2.90-5.92).

Attitudes toward data security also played a role. Participants who expressed fewer concerns about data security (somewhat agree that I am concerned about data security vs completely agree: aOR 1.46, 95% CI 1.11-1.93; neither agree nor disagree vs completely agree: aOR 1.37, 95% CI 1.03-1.82; somewhat disagree vs completely agree: aOR 1.76, 95% CI 1.35-2.33; strongly disagree vs completely agree: aOR 1.76, 95% CI 1.35-2.31) had higher odds of DHI uptake. Additionally, a stronger belief in the benefits of digital services (aOR 1.04, 95% CI 1.02-1.05) was associated with increased DHI uptake. Finally, individuals who reported good connections had the highest estimated odds of adopting the DHI.

**Table 8:** Logistic regression and LRT^a^ results for e-service^b^ use–related predictor variables.

Predictor variables		LRT		Logistic regression	
		*χ*^2^ (*df*)	FDR^c^-adjusted *P* value	aOR^d^ (95% CI)	*P* value (group comparison)
**Independence of e-service use**		**70.22 (2)**	**<.001**	—^e^	—
	Do not use (reference)	—	—	—	—
	Use with help or someone else uses on my behalf	—	—	2.48 (1.19-5.47)	.02
	Use independently	—	—	6.08 (3.46-11.92)	<.001
**Competence to use online services**		**97.61 (3)**	**<.001**	—	—
	No or low competence (reference)	—	—	—	—
	Moderate competence	—	—	2.00 (1.44-2.85)	<.001
	High competence	—	—	3.49 (2.50-4.98)	<.001
	Very high competence	—	—	4.10 (2.90-5.92)	<.001
**E-services not accessible to me**		**6.41 (2)**	**.06**	—	—
	Completely or somewhat agree (reference)	—	—	—	—
	Neither agree nor disagree	—	—	1.03 (0.81-1.31)	.83
	Strongly or somewhat disagree	—	—	1.22 (0.98-1.52)	.08
**Concerned about data security**		**24.75 (4)**	**<.001**	—	—
	Completely agree (reference)	—	—	—	—
	Somewhat agree	—	—	1.46 (1.11-1.93)	.007
	Neither agree nor disagree	—	—	1.37 (1.03-1.82)	.03
	Somewhat disagree	—	—	1.76 (1.35-2.33)	<.001
	Strongly disagree	—	—	1.76 (1.35-2.31)	<.001
**Poor internet connections**		**11.11 (2)**	**.007**	—	—
	Completely or somewhat agree (reference)	—	—	—	—
	Neither agree nor disagree	—	—	0.91 (0.70-1.20)	.51
	Strongly or somewhat disagree	—	—	1.22 (0.98-1.53)	.07
Benefits of digital services		27.38 (1)	<.001	1.04 (1.02-1.05)	—

^a^LRT: likelihood ratio test.

^b^e-service: electronic service.

^c^FDR: false discovery rate.

^d^aOR: adjusted odds ratio.

^e^Not applicable.

## Discussion

### Principal Findings

This study examined the relationship between background characteristics in four different domains (socioeconomics, health, lifestyle habits, and use of e-services) and the uptake of a lifestyle DHI (BitHabit). Overall, 18.2% (n=1287) of participants who received the invitation started using the BitHabit app. Women, middle-aged individuals, and those with higher income and education levels were more likely to take up the DHI. Moreover, better lifestyle choices as well as better functional capacity and current health were positively associated with higher odds of DHI uptake. Our research also underscored the impact of digital literacy and positive attitudes towards online services, revealing that individuals with greater familiarity and more positive views about online services were more likely to register for the DHI. On the contrary, men and individuals with lower socioeconomic status, limited digital skills, and poorer health and lifestyle habits were less likely to engage with the DHI. These findings imply that when offering DHIs, it may be challenging to engage those individuals who would benefit the most from a lifestyle DHI, emphasizing the importance of tailoring recruitment and intervention methods accordingly.

### Comparison With Prior Work

Although there is a scarcity of studies directly comparing DHI adopters and nonadopters, several studies exploring health app usage report that women and individuals in the young or middle-aged categories use more health apps [6,13,33,34]. These findings are also supported by recent results from a large population-level study comparing the characteristics of individuals who registered to use a mobile health physical activity intervention (N=696,907) with the entire population of Singapore; the results showed that women and younger participants are more inclined to register for a health app [14]. Our finding that women and those aged 35-49 years are more likely to take up a DHI is mainly consistent with previous studies examining health app usage. Interestingly, our results do not support the idea that young adults are more likely to take up DHIs. These conflicting findings could be due to differences in the study design, as most of the previous studies examining usage have focused on consumer-targeted health apps, which may be more appealing to younger adults.

Regarding income and education, previous studies have suggested that individuals with higher income levels or socioeconomic status and those with higher levels of education tend to use more health apps [6,13,15,16]. Our results showing that those with higher income levels and higher education are more likely to take up a DHI align with the previous findings on health app usage. This suggests that socioeconomic factors may continue to play a role in shaping the adoption of DHIs, highlighting the importance of considering these factors in future implementation strategies. However, although our study sheds light on who is more likely to adopt a DHI, it provides limited insight into the reasons why some individuals choose not to adopt DHIs. A deeper understanding of nonadopters is essential for designing inclusive digital interventions. Future research should explore the motivations and contextual factors that influence nonparticipation in DHIs.

DHIs and digital lifestyle interventions aim to promote healthy lifestyles and improve health outcomes. However, in general, it is not well known whether an individual’s lifestyle or health influences DHI uptake. Our results suggest that healthier lifestyle choices, such as better diet quality and nonsmoking behavior, along with better self-reported functional capacity and current health status, are associated with higher odds of DHI uptake. In this study, perceived lack of sleep was not associated with uptake of the BitHabit DHI; however, those individuals who were not sure of whether they got enough sleep had lower odds of taking up the DHI. This response might indicate a lack of personal interest in the individual’s lifestyle habits. However, it is challenging to draw strong conclusions based on this answer. Prior studies imply a potential link between health app usage and lifestyle choices, but results regarding diet quality, smoking, and physical activity still remain inconclusive in earlier investigations [13,15,17,18,35]. Conflicting results have also been found on the link between health app use and the BMI [14,15,18], as well as between chronic diseases and health app possession [15,17,18,36]. These earlier contradictory findings may be due to variations in the study design. Interestingly, those with better self-reported health status have been reported to be more likely to download health apps [36]. Overall, the results of this study regarding lifestyle habits and health imply that if a DHI aims to target those with poorer health and a nonhealthy lifestyle, we may not get the intended target population involved when using a “one-size-fits-all” recruitment strategy.

Digital literacy, defined as individuals’ ability to use digital technologies and understand their associated risks, emerged as a key factor in the uptake of the BitHabit DHI [37]. Our findings indicate that digital competence and views about online services are associated with the uptake of DHIs. In particular, individuals’ competence and their ability to use e-services independently are closely linked to their likelihood of taking up DHIs. Those reporting very high competence in using e-services had four times the odds to register for the DHI compared to those with little to no competence. Additionally, individuals who independently used e-services had six times the odds to take up the DHI compared to those who did not use e-services at all. These results highlight the importance of addressing the needs of individuals with limited e-service competence to enable them to access DHIs or of exploring alternative approaches. Furthermore, concerns about data security were associated with DHI uptake. Previous research on digital health services [19,38] and mobile health apps [20,21] has also highlighted the role of data security concerns as potential barriers to the usage of these services. Addressing security-related issues, such as ensuring compliance with regulations and enhancing transparency regarding data collection and handling, may help alleviate some of these concerns.

### Strengths and Limitations

Our approach has some strengths. Most importantly, our study population was drawn from participants of a recent population-based survey that included a wide age and socioeconomic distribution. The available knowledge of the background characteristics of the entire invited population enabled a direct comparison between adopters and nonadopters, instead of only studying adopters, which has been a common approach in earlier research.

There are also some potential limitations. It is worth noting that individuals who chose not to participate in the Healthy Finland survey were excluded from the sample used in the study. In addition, participants were asked to first fill the Healthy Finland survey online, which could introduce bias among respondents who were later approached and offered the DHI. Nevertheless, this bias may have been mitigated by the option to request a paper format for the survey. However, this potential bias may have led to a slight overrepresentation of digitally literate individuals, which could influence the generalizability of the findings to less digitally engaged populations. In addition, there was a 2-4–month delay between baseline data collection and app uptake, which may have allowed changes in predictors. This study was conducted in a single country (Finland), and participation was limited to Finnish-speaking individuals due to practical constraints, which may limit generalizability to other countries and language minorities within Finland. Specifically, the significance of the quality of internet connections in the area may be more pronounced in regions with a higher proportion of individuals experiencing poor connections. Finally, we did not collect explicit refusal data from nonparticipants. As such, we cannot distinguish between active refusal, missed invitations, or disinterest, which may affect the interpretation of nonuptake.

### Conclusion

DHIs have significant potential to deliver services to large population groups and thereby decrease the burden of NCDs at the population level. Despite this potential, there are notable concerns about unequal access to and use of DHIs and how this will affect health equity [39]. Our findings indicate that those with lower socioeconomic status, poorer digital literacy, and poorer health and lifestyle habits are less likely to take up DHIs. Therefore, effectively reaching individuals likely to benefit most from lifestyle DHIs may be complex. Consequently, it is important consider the targeted population group when designing recruitment strategies and intervention modalities. Otherwise, the digitalization of lifestyle interventions might lead to widening health equity gaps between population groups.

## Appendix

Multimedia Appendix 1 Questions on predictor variables in the Healthy Finland survey and diet quality scoring.

Multimedia Appendix 2 STROBE (Strengthening the Reporting of Observational Studies in Epidemiology) checklist for cross-sectional studies.

## Abbreviations

aOR: adjusted odds ratio
DHI: digital health intervention
e-service: electronic service
FDR: false discovery rate
LRT: likelihood ratio test
NCD: noncommunicable disease

## References

[1] Mathers CD, Loncar D (2006). Projections of global mortality and burden of disease from 2002 to 2030. PLoS Med.

[2] (2014). World Health Organization global status report on noncommunicable diseases 2014. World Health Organization.

[3] (2021). Discussion paper for the regional expert consultaitons – development of an implementation roadmap 2023–2030 for the global action plan for the prevention and control of NCDs 2013–2030. World Health Organization.

[4] Ford ES, Bergmann MM, Kröger J, Schienkiewitz A, Weikert C, Boeing H (2009). Healthy living is the best revenge: findings from the European prospective investigation into cancer and nutrition–Potsdam study. Arch Intern Med.

[5] O'Connor S, Hanlon P, O'Donnell CA, Garcia S, Glanville J, Mair FS (2016). Understanding factors affecting patient and public engagement and recruitment to digital health interventions: a systematic review of qualitative studies. BMC Med Inform Decis Mak.

[6] Szinay D, Jones A, Chadborn T, Brown J, Naughton F (2020). Influences on the uptake of and engagement with health and well-being smartphone apps: systematic review. J Med Internet Res.

[7] Di Cesare M, Khang Y, Asaria P, Blakely T, Cowan MJ, Farzadfar F, Guerrero R, Ikeda N, Kyobutungi C, Msyamboza KP, Oum S, Lynch JW, Marmot MG, Ezzati M, Lancet NCD Action Group (2013). Inequalities in non-communicable diseases and effective responses. Lancet.

[8] Sommer I, Griebler U, Mahlknecht P, Thaler K, Bouskill K, Gartlehner G, Mendis S (2015). Socioeconomic inequalities in non-communicable diseases and their risk factors: an overview of systematic reviews. BMC Public Health.

[9] Veinot T, Mitchell H, Ancker J (2018). Good intentions are not enough: how informatics interventions can worsen inequality. J Am Med Inform Assoc.

[10] Chatterjee A, Prinz A, Gerdes M, Martinez S (2021). Digital interventions on healthy lifestyle management: systematic review. J Med Internet Res.

[11] Michie S, Yardley L, West R, Patrick K, Greaves F (2017). Developing and evaluating digital interventions to promote behavior change in health and health care: recommendations resulting from an international workshop. J Med Internet Res.

[12] Murray E, Hekler EB, Andersson G, Collins LM, Doherty A, Hollis C, Rivera DE, West R, Wyatt JC (2016). Evaluating digital health interventions: key questions and approaches. Am J Prev Med.

[13] Carroll JK, Moorhead A, Bond R, LeBlanc WG, Petrella RJ, Fiscella K (2017). Who uses mobile phone health apps and does use matter? A secondary data analytics approach. J Med Internet Res.

[14] Yao J, Lim N, Tan J, Matthias Müller A, Martinus van Dam R, Chen C, Tan CS, Müller-Riemenschneider F (2022). Evaluation of a population‐wide mobile health physical activity program in 696 907 adults in Singapore. J Am Heart Assoc.

[15] Shen C, Wang MP, Chu JT, Wan A, Viswanath K, Chan SSC, Lam TH (2017). Health app possession among smartphone or tablet owners in Hong Kong: population-based survey. JMIR Mhealth Uhealth.

[16] Xie Z, Nacioglu A, Or C (2018). Prevalence, demographic correlates, and perceived impacts of mobile health app use amongst Chinese adults: cross-sectional survey study. JMIR Mhealth Uhealth.

[17] Ernsting C, Dombrowski SU, Oedekoven M, O Sullivan JL, Kanzler M, Kuhlmey A, Gellert P (2017). Using smartphones and health apps to change and manage health behaviors: a population-based survey. J Med Internet Res.

[18] Stühmann LM, Paprott R, Heidemann C, Baumert J, Hansen S, Zahn D, Scheidt-Nave C, Gellert P (2020). Health app use and its correlates among individuals with and without type 2 diabetes: nationwide population-based survey. JMIR Diabetes.

[19] Jokisch MR, Schmidt LI, Doh M (2022). Acceptance of digital health services among older adults: findings on perceived usefulness, self-efficacy, privacy concerns, ICT knowledge, and support seeking. Front Public Health.

[20] Wang C, Qi H (2021). Influencing factors of acceptance and use behavior of mobile health application users: systematic review. Healthcare (Basel).

[21] Zhou L, Bao J, Watzlaf V, Parmanto B (2019). Barriers to and facilitators of the use of mobile health apps from a security perspective: mixed-methods study. JMIR Mhealth Uhealth.

[22] Klaver NS, van de Klundert J, van den Broek RJGM, Askari M (2021). Relationship between perceived risks of using mHealth applications and the intention to use them among older adults in the Netherlands: cross-sectional study. JMIR Mhealth Uhealth.

[23] Pujia C, Ferro Y, Mazza E, Maurotti S, Montalcini T, Pujia A (2025). The role of mobile apps in obesity management: systematic review and meta-analysis. J Med Internet Res.

[24] Park Y, Tak YW, Kim I, Lee HJ, Lee JB, Lee JW, Lee Y (2024). User experience and extended technology acceptance model in commercial health care app usage among patients with cancer: mixed methods study. J Med Internet Res.

[25] Lundqvist A, Tolonen H (2023). Healthy Finland survey as the latest uptake for 50 years of population health monitoring in Finland. Eur J Public Health.

[26] Sääksjärvi K, Elonheimo H, Ikonen J, Lehtoranta L, Parikka S, Härkänen T, Vihervaara TI, Sainio P, Jääskeläinen T, Suominen AL, Harjunmaa U, Mäkelä P, Lahti J, Kaartinen NE, Koskinen S, Lundqvist A (2024). Cohort profile: Healthy Finland survey. Int J Epidemiol.

[27] Harjumaa M, Absetz P, Ermes M, Mattila E, Männikkö R, Tilles-Tirkkonen T, Lintu N, Schwab U, Umer A, Leppänen J, Pihlajamäki J (2020). Internet-based lifestyle intervention to prevent type 2 diabetes through healthy habits: design and 6-month usage results of randomized controlled trial. JMIR Diabetes.

[28] Lavikainen P, Mattila E, Absetz P, Harjumaa M, Lindström J, Järvelä-Reijonen E, Aittola K, Männikkö R, Tilles-Tirkkonen T, Lintu N, Lakka T, van Gils M, Pihlajamäki J, Martikainen J (2022). Digitally supported lifestyle intervention to prevent type 2 diabetes through healthy habits: secondary analysis of long-term user engagement trajectories in a randomized controlled trial. J Med Internet Res.

[29] (2023). Weekly physical activity recommendations. UKK Institute.

[30] Lindström J, Aittola K, Pölönen A, Hemiö K, Ahonen K, Karhunen L, Männikkö R, Siljamäki-Ojansuu U, Tilles-Tirkkonen T, Virtanen E, Pihlajamäki J, Schwab U (2021). Formation and validation of the Healthy Diet Index (HDI) for evaluation of diet quality in healthcare. Int J Environ Res Public Health.

[31] (2023). A language and environment for statistical computing. R Foundation for Statistical Computing.

[32] von Elm E, Altman DG, Egger M, Pocock SJ, Gøtzsche PC, Vandenbroucke JP, STROBE Initiative (2007). Strengthening the Reporting of Observational Studies in Epidemiology (STROBE) statement: guidelines for reporting observational studies. BMJ.

[33] Goyal S, Morita PP, Picton P, Seto E, Zbib A, Cafazzo JA (2016). JMIR Mhealth Uhealth.

[34] Paradis S, Roussel J, Bosson J, Kern J (2022). Use of smartphone health apps among patients aged 18 to 69 years in primary care: population-based cross-sectional survey. JMIR Form Res.

[35] Coughlin JW, Martin LM, Zhao D, Goheer A, Woolf TB, Holzhauer K, Lehmann HP, Lent MR, McTigue KM, Clark JM, Bennett WL (2022). Electronic health record–based recruitment and retention and mobile health app usage: multisite cohort study. J Med Internet Res.

[36] Robbins R, Krebs P, Jagannathan R, Jean-Louis G, Duncan DT (2017). Health app use among US mobile phone users: analysis of trends by chronic disease status. JMIR Mhealth Uhealth.

[37] Arias López MDP, Ong BA, Borrat Frigola X, Fernández AL, Hicklent RS, Obeles AJT, Rocimo AM, Celi LA (2023). Digital literacy as a new determinant of health: a scoping review. PLOS Digit Health.

[38] Foley K, Freeman T, Ward P, Lawler A, Osborne R, Fisher M (2021). Exploring access to, use of and benefits from population-oriented digital health services in Australia. Health Promot Int.

[39] Iyamu I, Gómez-Ramírez O, Xu AX, Chang H, Watt S, Mckee G, Gilbert M (2022). Challenges in the development of digital public health interventions and mapped solutions: findings from a scoping review. Digit Health.

